# Topology optimization of adaptive sandwich plates with magnetorheological core layer for improved vibration attenuation

**DOI:** 10.1177/10996362241278231

**Published:** 2024-08-27

**Authors:** Maryam Zare, Ramin Sedaghati

**Affiliations:** Department of Mechanical, Industrial and Aerospace Engineering, 5618 Concordia University, Montreal, QC, Canada

**Keywords:** Magnetorheological elastomer, sandwich plate, topology optimization, vibration attenuation, dynamic compliance

## Abstract

In this study the optimum topology distribution of the magnetorheological elastomer (MRE) layer in an adaptive sandwich plate is investigated. The adaptive sandwich plate consists of an MR elastomer layer embedded between two thin elastic plates. A finite element model has been first formulated to derive the governing equations of motion. A design optimization methodology incorporating the developed finite element model has been subsequently developed to identify the optimum topology treatment of the MR layer to enhance the vibration control in wide-band frequency range. For this purpose, the dynamic compliance and density of each element are defined as the objective function and design variables in the optimization problem, respectively. The method of the solid isotropic material with penalization (SIMP), is extended for material properties interpolation leading to a new MRE-based penalization (MREP) model. Method of moving asymptotes (MMA) has been subsequently utilized to solve the optimization problem. The developed finite element model and design optimization method are first validated using benchmark problems. The proposed design optimization methodology is then effectively utilized to investigate the optimal topologies of the magnetorheological elastomer (MRE) core layer in MRE-based sandwich plates under various boundary and loading conditions. Results show the effectiveness of the proposed design optimization methodology for topology optimization of MRE-based sandwich panels to mitigate the vibration in wide range of frequencies.

## Introduction

Structural vibration control is a promising method for mitigating the detrimental effects of excessive vibration in structures. It involves monitoring the dynamic behavior of a structure and implementing control strategies to reduce the vibration levels of the structure. Among various control methodologies, the semi-active control method adeptly combines the reliability characteristic of passive systems with the adaptability inherent in fully active systems, without requiring complex control hardware. Smart materials play a crucial role in implementing semi-active vibration control, and among them, magnetorheological (MR) materials^1–5^ have garnered substantial attention for their remarkable properties, including fail-safe feature, rapid response times (less than milliseconds) and low energy consumption. Compared with MR fluids (MRFs) which can only provide variable damping, magnetorheological elastomers (MREs) have field dependent viscoelastic properties in which both stiffness and damping properties can be effectively altered using the applied magnetic field.^6,7^

By incorporating MREs into the core of sandwich plates, it becomes possible to design an adaptive continuous sandwich structures which their vibration characteristics can be altered through application of an external magnetic field. There are a number of some studies addressing the dynamic characteristics of MRE-based sandwich structures, wherein an array of various modeling techniques and experimental approaches have been developed and reported. Nayak et al.^8^ evaluated the forced vibration of an MRE-cored sandwich beam. In their study, a magnetorheological elastomer (MRE) was fabricated with carbonyl iron particles mixed with silicon rubber as the matrix. The sandwiched beam consisted of an MRE core layer sandwiched between two thin elastic layers. They conducted experimental tests to investigate the effect of a magnetic field on the vibration response of the sandwich beam and concluded that the stiffness and damping the MRE based sandwich beam are substantially changed under application of an external magnetic field. Babu and Vasudevan^9^ conducted an investigation into the dynamic performance of a tapered laminated composite sandwich plate fully treated with MRE core layer. They also formulated the finite element (FE) model using classical limited plate theory and validated the model experimental tests. In another study, Vemuluri et al.^10^ investigated the dynamic characteristics a tapered laminated composite sandwich plate partially treated with MRE core layer. They subsequently investigated the effect of location and size of MRE segments on MRE based tapered laminated sandwich plate with different boundary conditions.

Choi et al.^11^ examined the dynamic characteristics of a smart sandwich beam with an MRE core layer. They improved an analytical model based on Frostig’s high-order beam theory to incorporate viscoelastic material properties and their frequency dependence while also exploring different boundary conditions and variations in the portions of the MRE core. The stochastic micro-vibration response of a clamped–free sandwich beam with an MR elastomer core was investigated by Ying et al.^12^ They derived the governing equation of motion for the sandwich beam by considering dynamic equilibrium, constitutive relations, and geometric factors. Additionally, they developed a frequency-domain solution method for analyzing the stochastic micro-vibration response, utilizing concepts of frequency-response functions, power spectral density functions, and spatial eigensolution. In recent studies, more complex sandwich plates incorporating MRE layers have been investigated. For instance, in a study conducted by Li et al.,^13^ the static and dynamic performance of MRE sandwich plates was examined, wherein the MRE core comprises two copper wire layers, two inner metal layers, and one MRE layer. Dynamic stability of a sandwich laminated composite plate fully treated with MRE core layer was investigated by Hosseinzadeh and Rezaeepazhand.^14^ The study examines how damping treatment and stability boundaries are influenced by different parameters, such as stacking sequences, boundary conditions, sandwich plate geometry, thickness, and partial activation of the MRE layer. Although complete coverage of the MRE core layer within a sandwich plate is likely to yield the best results in reducing vibration levels, it is essential to consider practical factors like mass limitations and activation of MRE in large surface area. Therefore, optimizing the topology of the MRE layer considering a constrained volume fraction is of practical importance.

Topology optimization is a design optimization method used to find the best distribution of material within a predefined domain, given a set of loads, constraints, and boundary conditions, in order to achieve optimal structural performance. Depending on the continuity of main domain, topology optimisation is classified into discrete and continuous types. In discrete topology optimization, the aim is to find the optimum number, positions and connectivity of structural members. Topology optimization methods for continuous structures can be broadly classified as: homogenization-based density-based, evolutionary-based methods. In a study conducted by Vemuluri et al.,^15^ the optimal locations of magnetorheological elastomer (MRE) segments within partially treated tapered composite MRE sandwich plates are investigated. Their study formulates an optimization problem with the aim of maximizing natural frequencies and loss factors, employing the finite element method in conjunction with a Genetic Algorithm (GA). Snamina^16^ conducted a study involving a rectangular sandwich plate with an MR fluid material as the core layer. They developed an optimization problem with the objective of identifying the optimal placement of active segments within the MRF layer based on evaluation of energy dissipated in the MRE layer.

Previous studies have primarily focused on determining the optimal locations of discrete MRE layers, without addressing the optimal topology distribution of MREs. In some studies, topology optimization problem has been formulated to maximize vibration suppression by identifying the best distribution of passive damping layers. In research conducted by Kang,^17^ reducing the residual vibration of shell structures under impact loads was investigated by optimizing the distribution of passive damping layer. In their optimization problem, an integrated square performance measure of residual vibration was used as the objective function, and an efficient adjoint method was employed to calculate the sensitivities. The topology optimization was based on widely used Solid Isotropic Material with Penalization Method (SIMP). Kim et al.,^18^ obtained the optimum distribution of damping layers in a shell structure for mitigating the vibration by maximization of the damping effect (the modal loss factor). Some research also attempted on achieving the optimum distribution of piezoelectric actuators in structures to optimize their performance. For instance, Guzmán et al.^19^ determined the optimal layout of piezoelectric transducers. In their approach, they aimed to maximize two objective functions: the trace of the controllability gramian matrix and the trace of the observability gramian matrix. The proposed optimization approach was based on a SIMP material interpolation model, a spatial filter, and the Sequential Linear Programming (SLP) method with moving limits. Homayouni-Amlashi et al.^20^ provided a Matlab code for topology optimization of piezoelectric actuators based on the Method of Moving Asymptotic (MMA). In their study, the extension of SIMP approach known as PEMAP-P (piezoelectric material with penalization and polarization) was used for the material interpolation. Noh et al.^21^ developed an optimization procedure to obtain the optimal layouts for piezoelectric energy harvesting devices (EHDs) considering the effect of static and harmonic dynamic mechanical loads. In their model, material properties including the anisotropic linear elasticity coefficients, piezoelectric coefficients, and permittivity coefficients were interpolated using the SIMP approach. Very limited study has been conducted on topology optimization of MR material as the core layer in sandwich plates. The only reported study is conducted by Zhang and Kang^22^ who investigated the topology optimization of the MR fluid core layer in a sandwich plate in an attempt to improve semi-active vibration performance. They used pseudo-densities as design variables to describe the MR fluid distribution while an artificial magneto-rheological fluid model (AMRF) with penalization was developed to remove intermediate density values.

In the present study, the overall goal is to develop a design optimization methodology for identifying the optimal distribution of the MRE core layer in a sandwich plate to achieve maximum reduction in vibration levels. The core of the structure comprises of an MRE layer sandwiched between two elastic thin plates. A finite element (FE) model has been developed using 4-node rectangular elements with 28 degrees of freedom to analyze the adaptive MRE-based sandwich plate structure. The developed FE model has then been effectively used to formulate an optimization problem to identify the optimal distribution of MRE core layer. Dynamic compliance and density of each MRE element are defined as the objective function and design variables, respectively. SIMP method is extended for material properties interpolation, resulting in the development of the MRE-based penalization (MREP) method. The optimization problem is subsequently solved using the MMA method, which is based on a special type of convex approximation.^23^ The effectiveness of the developed finite element model and topology optimization formulation are validated with those available in the literature. The developed design optimization methodology was then effectively utilized to to investigate the optimal topologies of MRE core layer in MRE-based sandwich plates under various boundary and loading conditions.

## Finite element modeling of the MRE sandwich plate

**Figure 1.**
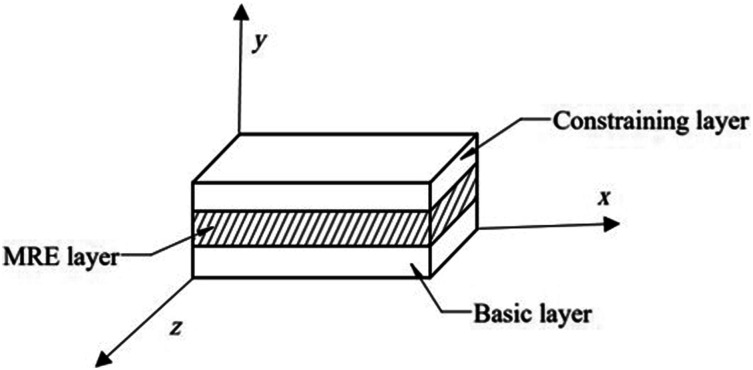
Schematic view of the MRE core sandwich plate.

The schematic view of the sandwich plate with MRE core layer is shown in Figure 1.

The sandwich plate structure comprises two elastic layers at the top and bottom, serving as constraining and base layers, respectively, with an isotropic MRE layer integrated as the core layer.

The FE model for the MRE based sandwich plate is developed based on the work of the work of Huang et al.^24^ and Yeh and Chen^25^ who formulated a FE model for the three-layer sandwich plate with passive viscoelastic core layer and ER core layer, respectively considering the following assumptions:

■ Kirchhoff plate theory is used for modeling the top and bottom layers. Thus, transverse shear strains and deformation through the thickness are ignored. It is assumed that there are no stretching and shear deformation in mid-plane of elastic layer except bending deformation.
■ The moment of the inertia of each layer is ignored.
■ The layers are considered completely bonded to each other without any slippage.
■ The MRE core operates under small shear deformation thus linear viscoelastic behavior is assumed.

**Figure 2.**
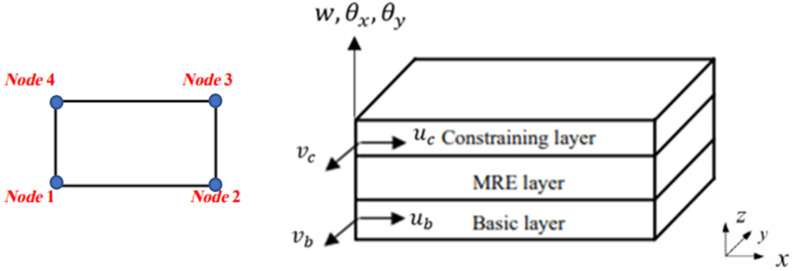
The schematic diagram of the 4-noded sandwich element.

Here, a three-layer sandwich rectangle element containing four nodes has been formulated to develop the FE model of the MRE-based sandwich plate. Each node possesses seven DOFs, including the displacement of the constraining and basic layers along the x and y directions, denoted as $u_{c},v_{c},u_{b},v_{b}$ , respectively, as well as the plate’s transverse deflection, w . Additionally, there are rotations about the x and y axes, represented as $\theta_{x},\theta_{y}$ , respectively. Figure 2 depicts a schematic diagram of the element, illustrating only the seven degrees of freedom of the first node for clarity.

The element displacement function can be expressed in the following vector form:

(1) $$W\left(t\right)={\left\{u_{c},v_{c},u_{b},v_{b},w,\theta_{x},\theta_{y}\right\}}^{T}$$

The nodal displacement vector may also be represented as:

(2) $${\;W}^{e}\left(t\right)={\left\{u_{ci},v_{ci},u_{pi},v_{pi},w_{i},\theta_{xi},\theta_{yi}\right\}}^{T}\;i=1,2,3,4$$

The element displacement vector can be expressed in terms of nodal displacement vector as:

(3) $$W=NW^{e}$$

where the 7 × 28 matrix N is the shape function matrix of the element relating the element displacement vector to the nodal displacement vector.

To derive the equations of motion for the element, the Hamilton’s principle is employed as:

(4) $$\delta \int_{t1}^{t2}\left(T-V\right)dt=0$$

here, T and V represent the kinetic and potential energy of the sandwich plate. The total kinetic energy of the MRE-based sandwich plate is the sum of the extension kinetic energy and bending kinetic energy associated with all layers. The kinetic energy of each layer, with a density denoted as ρ, is defined as:

(5) $$T=\frac{1}{2}\rho {∭}_{V}\left({\left(\frac{\partial u}{\partial t}\right)}^{2}+{\left(\frac{\partial v}{\partial t}\right)}^{2}+{\left(\frac{\partial w}{\partial t}\right)}^{2}\right)dV$$

The potential energy corresponding to each layer consists of two parts: bending potential energy which is associated with the deformation of the plate due to bending, and the tensile potential energy, associated with the deformation of the plate due to tensile stresses. The potential energy of each of the elastic layers (the constraining and basic layer), with the tensile and bending stresses of $\sigma_{T}$ and $\sigma_{b}$ , and the corresponding strain of $\varepsilon_{T}$ and $\varepsilon_{b}$ is calculated as:

(6) $$V=\frac{1}{2}{∭}_{V}\left({\varepsilon_{T}}^{T}\sigma_{T}+{\varepsilon_{b}}^{T}\sigma_{b}\right)dV$$

For the MRE layer, its potential energy encompasses tensile, bending, and shear strain energies, which are expressed as:

(7) $$V=\frac{1}{2}{∭}_{V}\left({\varepsilon_{T}}^{T}\sigma_{T}+{\varepsilon_{b}}^{T}\sigma_{b}+\gamma^{T}G\;\gamma \right)dV$$

where $\gamma ={\left[\gamma_{x}\;\gamma_{y}\right]}^{T}$ is the shear starin of MRE layer, and G is the complex shear stiffness modulus. Assuming isotropic MRE, $G_{x}=G_{y}=G_{MRE}$ , we can write:

(8) $$G=\left[\begin{array}{ll} G_{MRE} & 0\\ 0 & G_{MRE} \end{array}\right]$$

in which $G_{MRE}$ is the complex shear modules of the MRE, which in linear viscoelastic region can be described as:

(9) $$G_{MRE}=G_{MRE}^{\prime }+iG_{MRE}^{\prime \prime }$$

where $G_{MRE}^{\prime }$ and $G_{MRE}^{\prime \prime }$ are field dependent storage modulus (stiffness) and loss modulus (damping) properties of the MRE. Substituting the strain and kinetic energies into Hamilton’s principle, the governing equation of the motion for the MRE-based sandwich plate element can be derived as:

(10) $$M^{e}\;{\ddot{W}}_{i}{\left(t\right)+K}^{e}W_{i}\left(t\right)={f_{i}}^{e}$$

where, ${f_{i}}^{e}$ represents the external force applied to the element and $M^{e}$ and $K^{e}$ are the mass and complex stiffness matrices of the element and their derivations are provided in Appendix A. Assembling the derived stiffness and mass matrices for all elements yields the system finite element formulation of the MRE-based sandwich plate as:

(11) $$M\;\ddot{W}+K^{*}\;W=F$$

where M and $K^{*}$ represent the assembled mass and stiffness matrices of the entire MRE-based sandwich plate respectively, while F is the vector representing external nodal forces. Additionally, W denotes the system nodal displacement vector of the sandwich plate.

It is noted that vibration and damping characteristics of the sandwich plate can be investigated using free vibration eigenvalue problem as:

(12) $$\left(K-{\omega^{*}}^{2}M\right)\;\bar{W}=0$$

where $\omega^{*}$ is the complex natural frequency. The square root of real part of ${\omega^{*}}^{2}$ and the ratio of its imaginary over the parts represent the natural frequency and damping factor of the sandwich plate.

**Figure 3.**
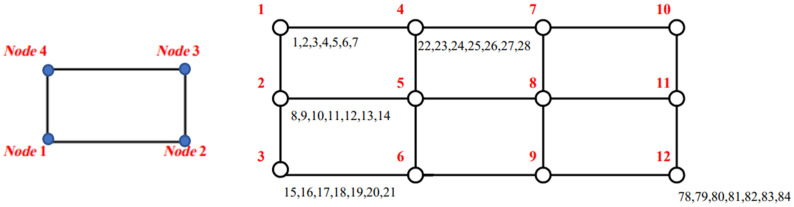
Finite element discretization of design domain.

Figure 3 illustrates the discretization view of the sandwich plate, along with the arrangement of node numbers used in this study.

## Topology optimisation problem

A topology optimization problem has been formulated to identify the leads for the optimal distribution of MRE core layer to maximize the semi-active vibration control performance of the MRE sandwich plate. There are generally five different major topology optimization methods.^26,27^ These include, topological derivative method, phase field method, evolutionary technique, level set method and density approach. The density approach was first developed by Bendsøe^28^ and has been widely used due to its effectiveness.^29^ The density-based approach has been effectively used in this study to develop the topology design optimization of the MRE core layer in the MRE based sandwich plate.

In this method, the material distribution is represented by a discretized domain, where each element corresponds to a design variable representing material density. Structural response, necessary for optimization, is typically computed using the finite element method, which also relies on domain discretization. In this context, the domain discretization used for density interpolation and finite element analysis is identical.

In theory, density can be represented as either zero or one, indicating the presence or absence of material in an element. However, this discrete approach is not suitable for many optimization methods. To address this issue, the density-based optimization approach is implemented to relax the optimization problem form the binary, on-off nature of the problem by defining a continuum density ranging between zero and one.^28^

### Material properties interpolation

In the density-based optimization approach, the material properties of each element in the discretized domain are related to their density by applying a power-law interpolation function. One of the methods used for the material interpolation scheme is SIMP method.^30^ The SIMP approach is widely regarded for its conceptual and practical simplicity and has proven successful in many applications.^31–34^ This approach involves discretizing the design domain into small elements and assigning a design variable (the element density) to each element. It is also assumed that the material properties within each element are constant and isotropic.^35^ For intermediate densities (i.e., densities between 0 and 1), the SIMP approach penalizes these intermediate densities using a penalization factor to achieve better binary topology patterns. Utilizing this method, the density ρ , and material property, E, are interpolated using an artificial model, as follows:

(13) $$\begin{array}{l} \rho \left(x_{i}\right)=x_{i}\;\rho \\ E\left(x_{i}\right)={\left(x_{i}\right)}^{p}\;E \end{array}$$

where $x_{e}$ is the relative density of each element and p is the penalization factor. It is noted that the density $\rho \left(x_{i}\right)$ is the element design function ranging between 0 at $x_{i}=0$ to material density ρ at $x_{i}=1$ .

For the present study, however, the vibration damping is mainly due to shear deformation of the MRE layer, and thus the shear modulus of MRE layer is interpolated based on the SIMP method. In the developed MRE-based penalization (MREP) model, the complex shear modulus $G_{MRE}$ , and the material density, ${\;\rho }_{MRE},$ of each MRE element are respectively defined as:

(14) $$\begin{array}{l} G_{MRE}\left(x_{i}\right)={\left(x_{i}\right)}^{p}G_{MRE}\\ \rho_{MRE}\left(x_{i}\right)=x_{i}{\;\rho }_{MRE} \end{array}$$

The value of the penalization factor p depends on the problem generally, although the value of 3 has been reported to provide a satisfactory solution.^30^

Considering the material interpolation functions describe in equation (14), and using the developed FE model, one can obtain the following expressions for the stiffness and mass matrices of the MRE layer elements as:

(15) $$\begin{array}{l} K_{MRE}\left(x_{i}\right)={\left(x_{i}\right)}^{p}\;K_{MRE}\\ M_{MRE}\left(x_{i}\right)=x_{i}\;M_{MRE} \end{array}$$

It is noted that, stiffness and mass matrices of the MRE layer elements varies from 0 at $x_{i}=0$ at to their full coverage value $K_{MRE}$ and $M_{MRE}$ , at $x_{i}=1,$ respectively.

### Formulation of the topology optimization problem

To formulate the optimization problem effectively, it is essential to define a suitable objective function that ensures the best performance of the structure in reducing the vibration level. In harmonic vibration problems, one of the most commonly used objective functions is dynamic compliance, first introduced by Ma et al.^36^ and initially defined as an extension of the concept of compliance used in static problems.^37^

The dynamic compliance of a sandwich plate, denoted as *f*, for a discretized domain may be described as:

(16) $$f=U^{T}F$$

where *F* and *U* represent the vector of nodal force and displacement degrees of freedom.

While applying a full coverage of MRE layer may provide maximum reduction in vibration, it is not practically realizable due to weight constraints and design of electromagnet system to cover the whole design space. In this study, a topology optimization method based on the SIMP method has been formulated under MRE volume constraint. The optimization problem for an MRE-based sandwich plate under a harmonic loading with frequency ω can be mathematically ate as:

Find relative density vector x as:

(17) $$to\;Minimize\;f_{0}\left(X\right)Subject\;to\;\left\{ \begin{array}{l} \left(K-\omega^{2}M\right)U=F\;\\ \overset{N_{e}}{\underset{i=1}{\sum }}x_{i}V_{i}\leq V_{0}\;\\ \;0\leq x_{i}\leq 1\;i=1,2,3,\ldots ,N_{e}\; \end{array}\right.$$

Where *M* and *K* represent the system mass and stiffness matrices of the MRE based sandwich plate. The optimization problem variables are density of the MRE layer in each element, denoted as the $x_{i}$ , within a domain discretized with $N_{e}$ as the number of elements. Here, $x_{i}=0$ and $x_{i}=1$ correspond to void and solid elements in i th element considering the MRE layer, respectively. Additionally, $V_{i}$ represents the volume of an element with full coverage of the MRE layer, while $V_{0}$ denotes the allowable volume fraction, which imposes a mass restriction on the sandwich plate.

#### Optimization algorithm

In this study, the method of moving average (MMA) has been effectively used to solve the optimization problem defined in equation (17). The MMA, developed by Svanberg,^38,39^ has been widely used for structural topology optimization problems.^40–42^ MMA is based on the solution of a convex quadratic subproblem. The updated version of MMA method presented in Ref. 39 is globally convergent. It is noted that the efficiency of MMA is strongly influenced by the asymptote and move limits.

**Figure 4.**
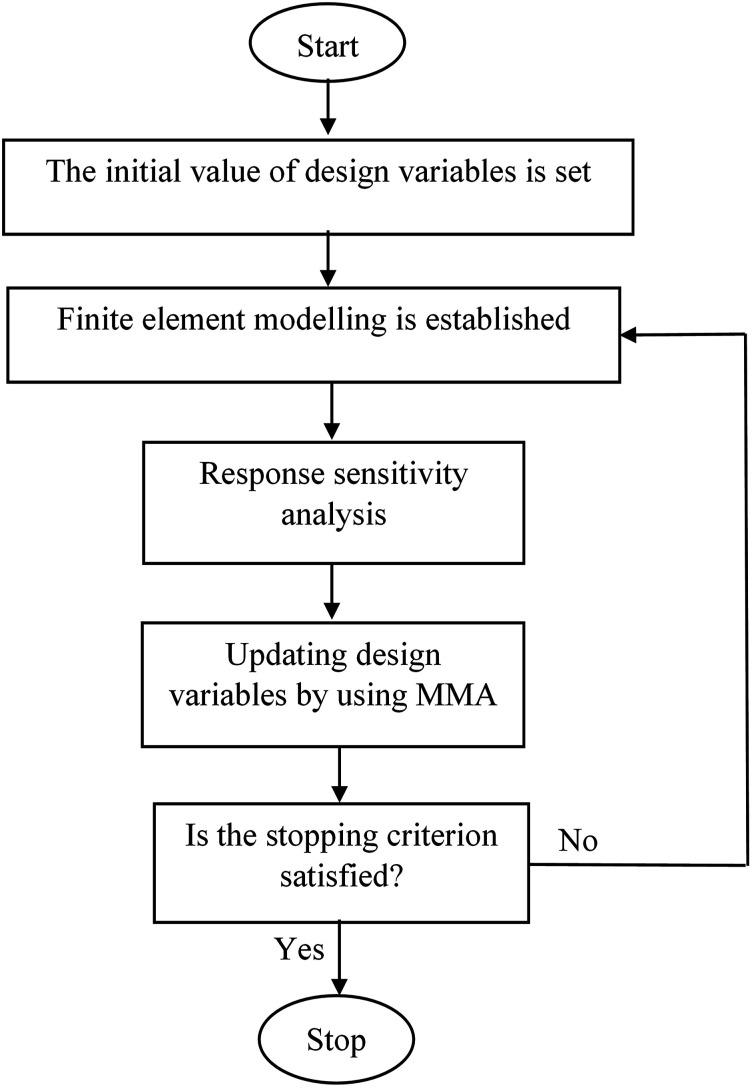
Flowchart of the proposed topology optimization method.

In MMA method, the structural optimization problem is generally cast into the following form^39^:

(18) $$\begin{array}{l} Minimise\;f_{0}\left(X\right)+a_{0}z+\overset{m}{\underset{i=1}{\sum }}\left(c_{i}y_{i}+\frac{1}{2}d_{i}{y_{i}}^{2}\right)\\ Subject\;to\;f_{i}\left(X\right)-a_{i}z-y_{i}\leq 0\;i=1,2,\ldots ..,c\\ x\in X,y\geq 0,z\geq 0 \end{array}$$

where, $f_{0}\left(X\right)$ is the cost function to be minimized and *X* is the vector of design variables, y and z are artificial optimization variables, c is the number of constraints and $a_{0}$ , $a_{i}$ , $c_{i}$ and $d_{i}$ are the constant coefficients. As stated in Ref. 39, setting $a_{0}=1$ and $a_{i}$ = 0 for all *i*, then z=0 in any optimal solution of optimization problem stated in equation (18), and also by considering $d_{i}=0$ and $c_{i}$ = “a large number,” subsequently the variables $y_{i}=0$ for all i in any optimal solution of equation (18). In this case, the optimization problem defined in equation (18) becomes equivalent to any general nonlinear optimization problem including that in equation (17). To solve the optimization problem, the external MMA code in Ref 42 has been used. The flowchart of the proposed topology optimization algorithm is shown in Figure 4.

It should be noted that the MMA necessitates the implementation of sensitivity analysis for the objective function (dynamic compliance). Given the complex nature of the stiffness matrix and the displacement vector, the dynamic compliance is defined as:

(19) $$C=U^{T}F=\left({U_{R}}^{T}+{U_{I}}^{T}i\right)\;\left({K_{R}}^{T}+{K_{I}}^{T}i-\omega^{2}M\right)\left(U_{R}+U_{I}i\right)$$

where, $U_{R}$ and $K_{R}$ represent the real parts of the stiffness matrix and displacement vector, while $U_{I}$ and $K_{I\;}$ represent their respective imaginary parts.

Using equation (19) and applying the chain rule, the sensitivity of dynamic compliance with respect to the design variable *x*_i_ is expressed as^43^:

(20) $$\begin{array}{l} \frac{\partial \left|C\right|}{\partial x_{j}}=\frac{1}{\left|C\right|}\left(C_{R}\frac{\partial C_{R}}{\partial x_{j}}+C_{I}\frac{\partial C_{I}}{\partial x_{j}}\right)\\ \frac{\partial C_{R}}{\partial x_{j}}={u_{R}}^{T}\left(\frac{\partial K_{R}}{\partial x_{j}}-\omega^{2}\frac{\partial M}{\partial x_{j}}\right)u_{R}-{u_{R}}^{T}\left(\frac{\partial K_{I}}{\partial x_{j}}\right)u_{I}-{u_{I}}^{T}\left(\frac{\partial K_{R}}{\partial x_{j}}-\omega^{2}\frac{\partial M}{\partial x_{j}}\right)u_{I}-{u_{I}}^{T}\left(\frac{\partial K_{I}}{\partial x_{j}}\right)u_{R}\\ \frac{\partial C_{I}}{\partial x_{j}}={u_{R}}^{T}\left(\frac{\partial K_{R}}{\partial x_{j}}-\omega^{2}\frac{\partial M}{\partial x_{j}}\right)u_{I}+{u_{R}}^{T}\left(\frac{\partial K_{I}}{\partial x_{j}}\right)u_{R}+{u_{I}}^{T}\left(\frac{\partial K_{R}}{\partial x_{j}}-\omega^{2}\frac{\partial M}{\partial x_{j}}\right)u_{R}-{u_{I}}^{T}\left(\frac{\partial K_{I}}{\partial x_{j}}\right)u_{I} \end{array}$$

Referring to equation (15), the following expressions are derived for the derivatives of the stiffness and mass matrices:

(21) $$\frac{\partial M}{\partial x_{i}}=M_{MRE}\;;\;\frac{\partial K}{\partial x_{i}}={p\left(x_{i}\right)}^{p-1}K_{MRE}$$

## Results and discussions

In this section, first the accuracy of the developed finite element (FE) model and topology optimization formulation are verified. The proposed design optimization method is then effectively used to investigate the optimal topologies of the magnetorheological elastomer (MRE) core layer in MRE-based sandwich plates under various boundary and loading conditions.

### Finite element model validation

In this section, the validity of the developed FE model executed in the Matlab environment is examined by considering a rectangular sandwich plate treated with a MR core layer. The sandwich plate is simply supported in all four edges and its properties are based on those reported by Yeh^44^  and Zhang^45^ summarized in Table 1. It noted in both Ref. 44 and Ref. 45 the properties of MR core layer is assumed to be of that of MR fluid.

**Table 1.** Mechanical and material properties of the sandwich plate^44,45^.

Top and bottom layers	Young’s modulus	70 Gpa
	Poison’s ratio	0.3
	Mass density	2700 kg/m^3^
	Constraining layer thickness	0.375 mm
	Base layer thickness	1.25 mm
MRE layer	Thickness	0.625 mm
	Mass density	3500 kg/m^3^
Plate dimensions	Length	0.3 m
	Width	0.2 m

The validity of the FE model is assessed by comparing the natural frequencies of the sandwich plate with those reported in Refs. 44,45. Table 2 provides results for the first four natural frequencies under two different values of the average magnetic flux density of 200 and 400 G. As can be realized, results are in very good agreement with those obtained by Yeh^44^ and Zhang.^45^

**Table 2.** Comparisons of the first four natural frequencies (Hz) of the MRE sandwich plate under two different magnetic flux densities.

Natural frequency	Present study (Hz)	Yeh^44^ (Hz)	Error%	Zhang and Kang^45^ (Hz)	Error%
Magnetic flux density (200G)					
First	108.28	107.34	0.88	109.39	1.02
Second	188.08	183.81	2.32	188.26	0.10
Third	284.04	283.06	0.35	283.67	0.13
Fourth	314.18	313.09	0.12	313.97	0.07

Natural frequency	Present study (Hz)	Yeh^44^ (Hz)	Error%	Zhang an Kang^45^ (Hz)	Error%
Magnetic flux density (400G)					
First	113.74	115.01	1.10	115.32	1.37
Second	197.14	195.21	0.98	197.03	0.06
Third	295.60	296.21	0.206	294.70	0.30
Fourth	326.95	327.15	0.06	325.49	0.45

### Topology optimization validation

**Figure 5.**
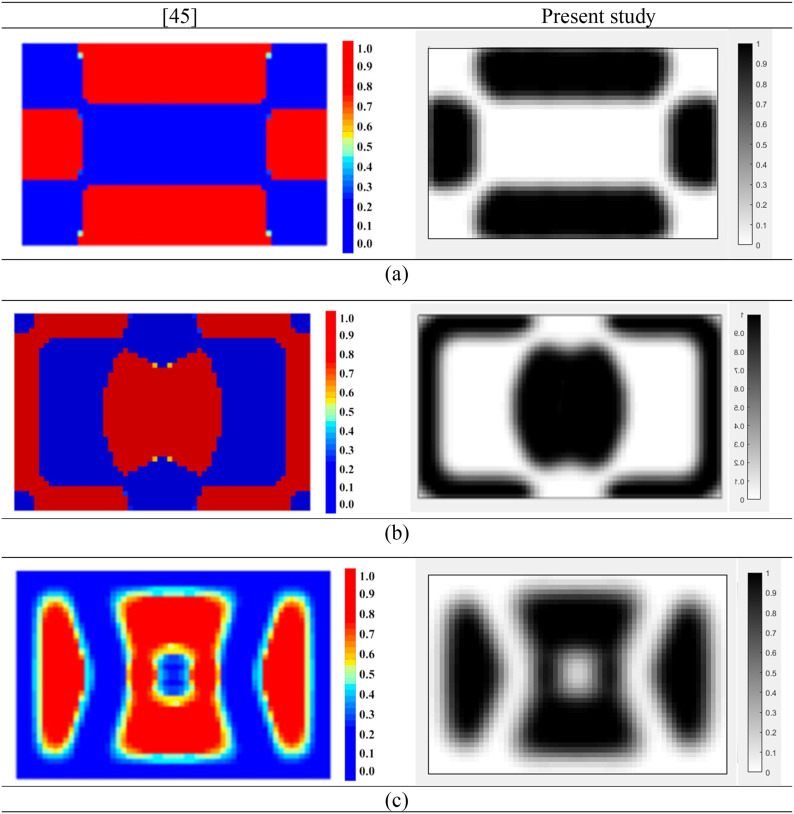
Comparisons of the optimization topology results for MR fluid core layer in different excitation frequencies. (a) 80 Hz; (b) 280 Hz; (c) 400 Hz.

In this section, the accuracy of the proposed optimization approach is investigated. A rectangular MR based sandwich plate with properties similar to those studied by Zhang et al.^45^ is considered. In their study, the goal was to obtain the optimal topology distribution of MR fluid layer. To achieve this, an optimization problem was formulated with the dynamic compliance of the sandwich plate as the objective function while incorporating a mass constraint. To solve the optimization problem, globally convergent method of moving asymptotes (GCMMA) was utilized. The material properties of the base layer and the constraining layer, as well as the structural dimensions, are the same as those reported in Table 1. For the MR fluid layer, the density is 3500 kg/m^3^, and for the sealant material, the Young’s modulus, Poisson’s ratio, density, and thickness are 0.22 GPa, 0.4, 1233 kg/m^3^, and 0.1 mm, respectively. The plate is simply supported at all edges and subjected to concentrated harmonic loading applied at the middle of the plate. Figure 5 illustrates a comparison of the optimum topologies for the plate under a magnetic field of 200 G and a loading magnitude of 1000 N at different frequencies. It is worth noting that, to the best of our knowledge, there is no study on the topology optimization of MRE layer in MRE-based sandwich plates. Thus, for the validation purposes instead the available results for topology optimization of MR fluid-based sandwich plate reported by Zhang and Kang^45^ were used.

Figure 5 illustrates optimal topology results for all frequencies using the proposed design optimization methodology and those reported in Ref. 45. As it can be realized the optimal topologies of MR fluid core layer identified using the proposed topology optimization methodology are in very agreement with those identified by Zhang and Kang .^45^

### Topology optimization examples

In this section, the validated analysis and topology optimization methods are effectively used to investigate the optimal topologies of the magnetorheological elastomer (MRE) core layer in MRE-based sandwich plates under various boundary and loading conditions including CFCF (two edges are clamped and the other two are free) and SSSS (all edges are simply supported). In all the investigated examples, the plate is subjected to a magnetic field of 200G. The geometric boundary conditions for the clamped and simply supported edges are given, such that:

(22) $$\;\begin{array}{l} Clamped\;u_{c}=v_{c}=u_{b}=v_{b}=w=\theta_{x}=\theta_{y}=0\\ Simply\;supported\;w=0 \end{array}$$

It is noted that for the all optimization problems, the optimization process begins with the initial values of the design variables set to ( $\;x_{i}=0.5)$ . The penalty factor for the optimization problems is set at p= 0.5 and the mass of MRE topology treatment is constraint to be half of the mass of MRE full coverage.

#### Simply supported plate (SSSS)

**Figure 6.**
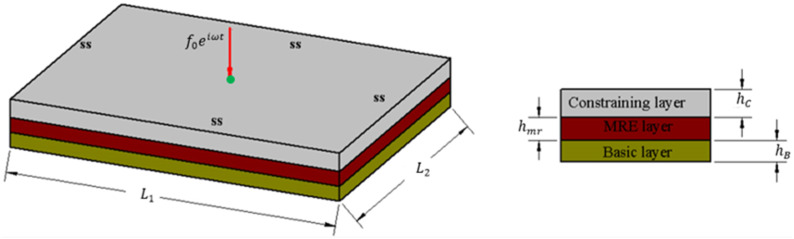
MRE-based sandwich plate (SSSS) under harmonic loading applied at the center.

A sandwich plate consisting of a MRE core embedded between two aluminum plates is considered (Figure 6). The sandwich plate has a dimension of $L_{1}=0.35$ m and $L_{2}=0.2$ m and its properties are summarized in Table 1. The plate is assumed to be simply supported at all four ends under a time-harmonic concentrated force ( $F=f_{0}e^{i\omega t}$ ) applied at the center of the plate. The magnitude, $f_{0},$ and the frequency, ω, of the loading are assumed to be 1000 N and 150 Hz, respectively.

The MRE core has a 25% volume fraction of carbonyl iron particles (CIPs) resulting in the core MRE density of 2771 kg/m³. It is assumed that MRE core layer operates in the linear viscoelastic region, thus the complex shear modulus $G_{MRE}$ of the MRE can be represented by equation (9).

For the considered MRE, the storage modulus can be determined as a polynomial function of the magnetic field intensity B (in Tesla) as reported in Ref. 46:

(23) $$G^{\prime }\left(B\right)=-1283B^{4}+1904B^{3}-271.2B^{2}+73.84B+55.19\;\left[kPa\right]$$

It is worth noting that, the storage modulus of the MRE changes significantly from 55 kPa in the absence of the applied magnetic field to saturation limit of nearly 479 kPa at magnetic flux density of 1 T. The change in loss factor with respect to applied magnetic field is found to be nearly negligible with average value of nearly 0.145.^46^

**Figure 7.**
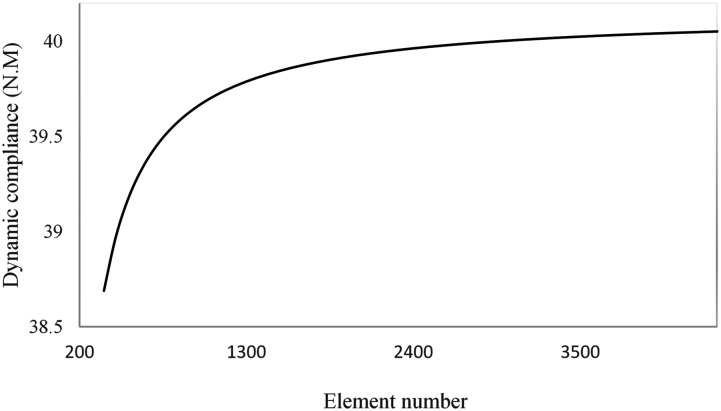
Convergence history of dynamic compliance.

To implement the finite element method, the convergence history of dynamic compliance is first analyzed to determine the optimal number of elements required for meshing the sandwich plate. Results shown in Figure 7 confirms that the variation in dynamic compliance decreases as the number of elements increases. It is note that increasing the number of elements beyond 3500 would have negligible effect on dynamic compliance. Considering the sandwich plate is meshed with (70 × 50) elements resulting in 24500 degrees of freedom.

**Figure 8.**
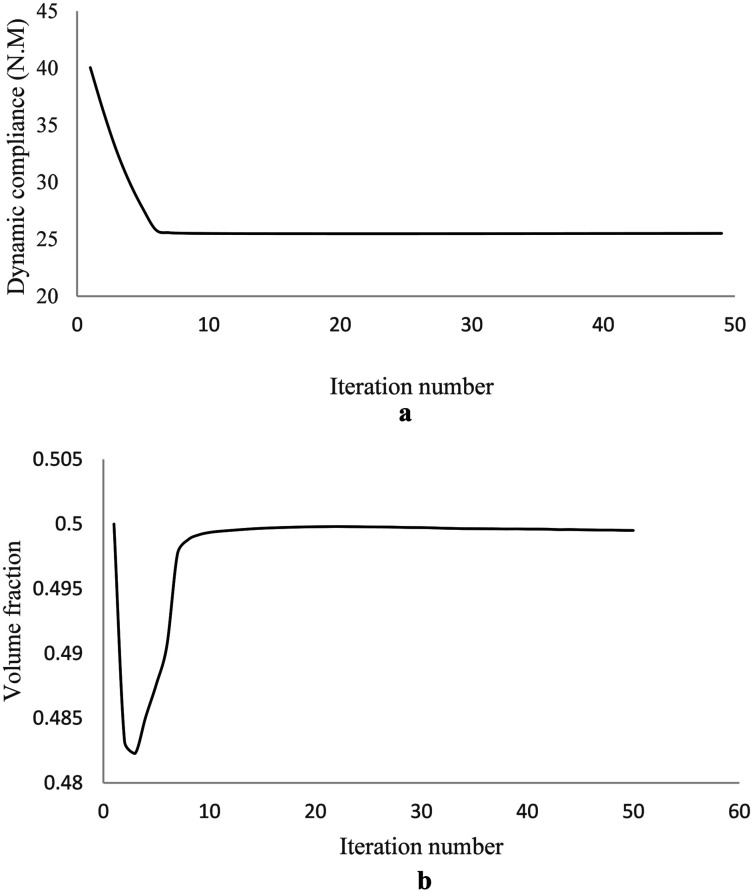
Optimization iteration history of the MRE sandwich plate (SSSS): (a) Convergence history of the dynamic compliance, (b) Convergence history of the volume fraction.

The optimization iteration history for the dynamic compliance and volume fraction are shown in Figure 8 suggesting rapid converge of the topology optimization algorithm.

**Figure 9.**
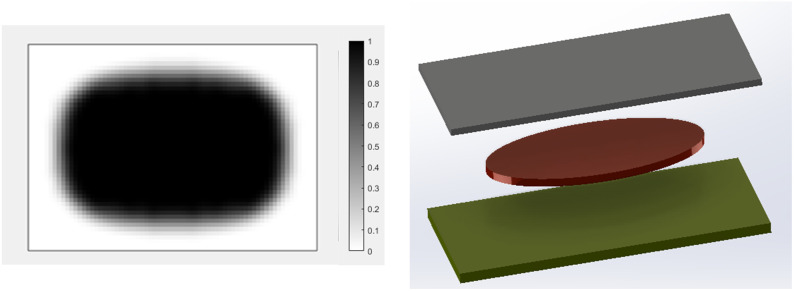
Optimal distribution of MRE layer for the MRE sandwich plate (SSSS) under the excitation frequency of ω=150 Hz.

**Figure 10.**
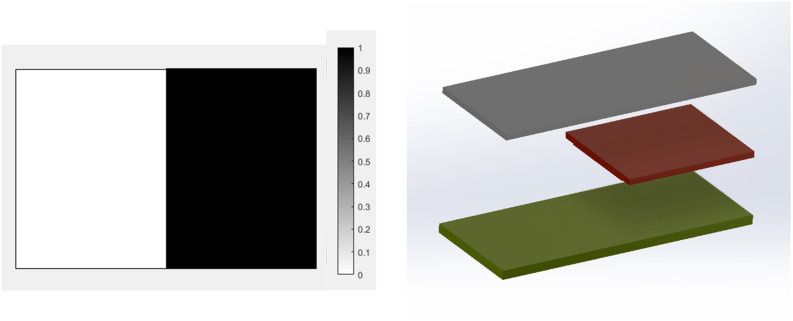
MRE distribution for an arbitrary case in the MRE sandwich plate (SSSS).

**Figure 11.**
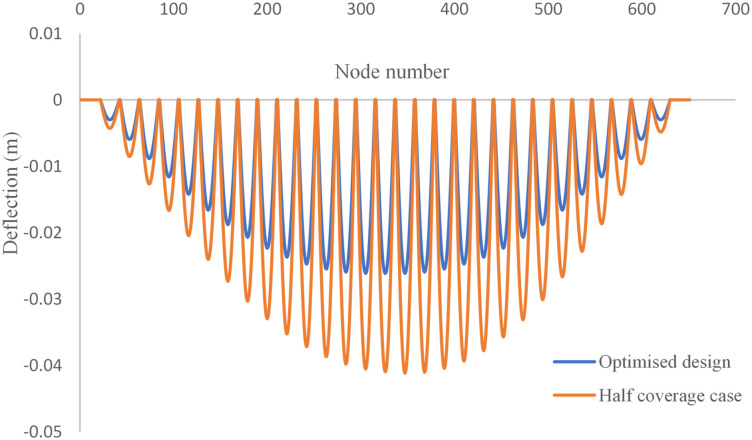
Optimal distribution of MRE layer for the MRE sandwich plate (SSSS) under the excitation frequency of ω=150 Hz.

Final optimal topology of the MRE layer is shown in Figure 9. As depicted in Figure 8, the dynamic compliance has decreased by almost 36.3% from f=40.05 N·m at initial configuration to f=25.5 1 N·m at the final optimum solution. The effectiveness of the identified optimum topology in reducing vibration level is compared with an arbitrary case shown in Figure 10 in which the entire MRE has been placed in one half of the plate (considering 50% volume fraction constraint on MRE). Results for vibration deflection amplitude at all nodes are shown in Figure 11. It is noted to that the numbering of nodes and their arrangements are based on the scheme shown in Figure 3. It should also be note that for the better demonstration and to include all nodes in Figure 11, the domain has been discretized using (30 × 20) elements. The results clearly show that the optimal topology configuration shown in Figure 9 significantly reduces the vibration amplitude compared with the MRE layout shown in Figure 10.

#### Clamped-clamped plate (CFCF)

**Figure 12.**
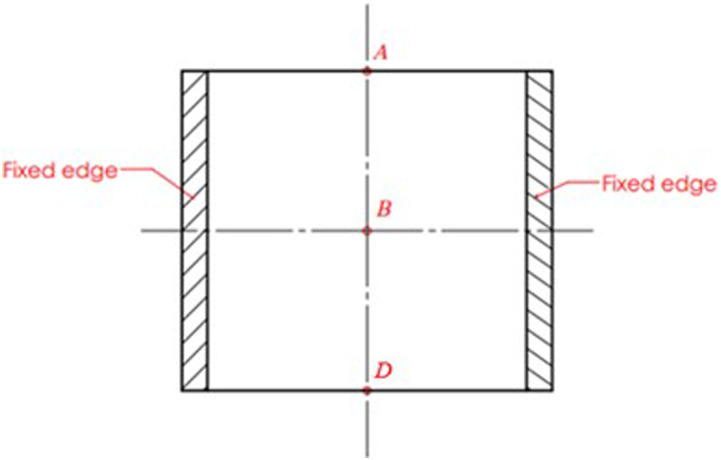
Clamped-clamped MR sandwich plate with loading positions of A.B and D.

In this example the optimal topology of the MRE layer in a square sandwich plate clamped ( $L_{1}=L_{2}=0.5$ m) at both opposite ends and free at the other two ends (Figure 12) is investigated. The properties of the constraining and basic layers, as well as the MRE layer and their thicknesses are the same as those of simply supported (SSS) sandwich plate presented in Table 3. It is noted that the plate is discretized using (60 × 60) elements.

**Table 3.** Mechanical and material properties of the MRE-based sandwich plate.

Top and bottom layers	Young’s modulus ( $E_{C},E_{B}$ )	70 Gpa
	Poison’s ratio ( $v_{C},v_{B}$ )	0.3
	Mass density ( $\rho_{C},\rho_{B}$ )	2700 kg/m^3^
	Constraining layer thickness ( $h_{C}$ )	0.4
	Base layer thickness ( $h_{B}$ )	2 (mm)
MRE layer	Thickness ( $h_{mr}$ )	0.4

The optimal topology of MRE layer has been investigated for two different cases as described below.

**Figure 13.**
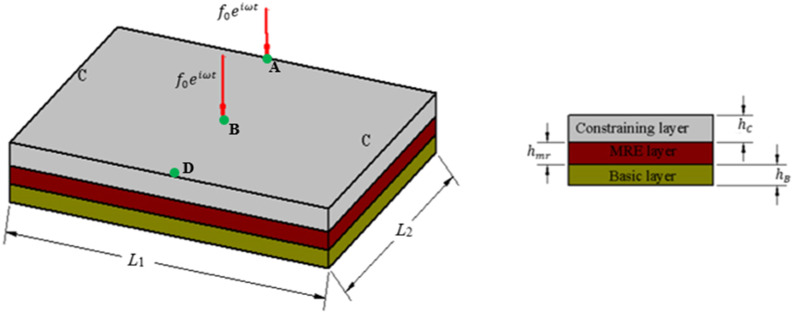
MRE sandwich plate (CFCF) with loading positions at A and B (Case I).

Case I In the first case, the MRE sandwich plate (CFCF) is under two concentrated harmonic loadings, one at the middle point B and the other at point A as shown in Figure 13. Both harmonic loads have magnitude and frequency of 1000 N and 280 Hz, respectively.

**Figure 14.**
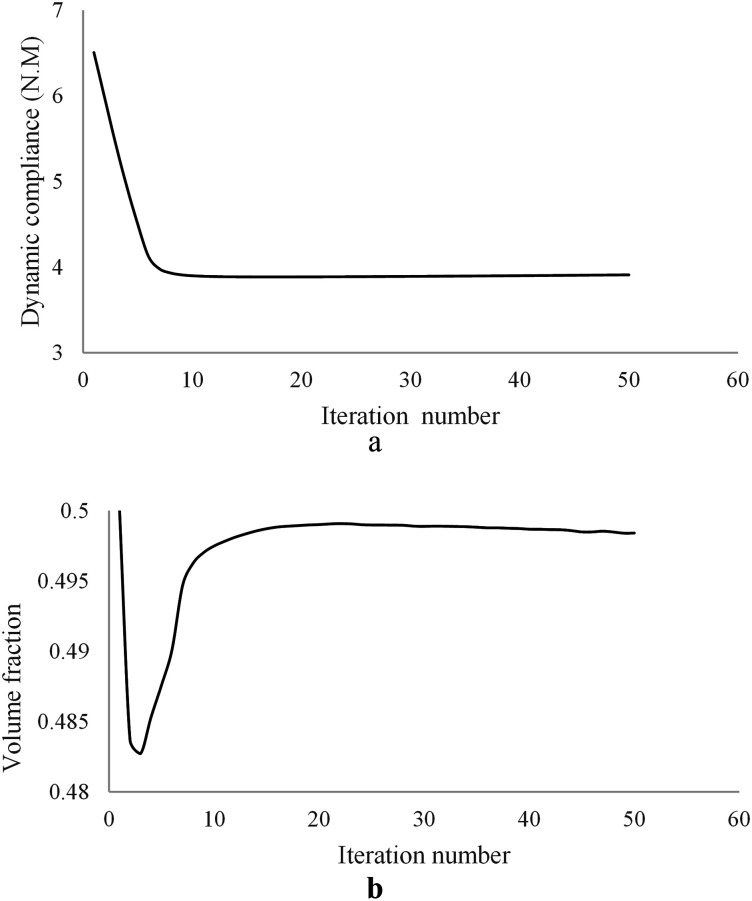
Optimization process history of MRE sandwich plate (CFCF): (a) Convergence history of the dynamic compliance, (b) Convergence history of the volume fraction (Case I).

For this case, the iteration history for the dynamic compliance and volume fraction is shown in Figure 14. Results show that the dynamic compliance has decreased by 34.8%, from f=6.50 N.m at the initial distribution to f=4.24 N.m at the optimum topology configuration.

**Figure 15.**
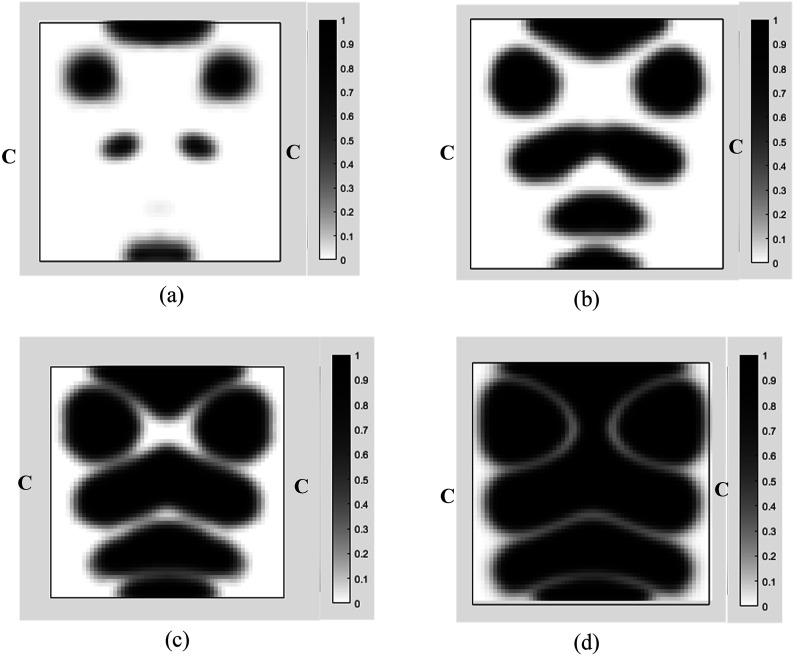
Optimal topology of the MRE layer for MRE sandwich plate (CFCF) under different volume constraints: (a) 0.1; (b) 0.3 (c) 0.5 (d) 0.7 (Case I).

The effect of volume fraction constraint on the optimum topology of the MRE has also been investigated and results for the volume fractions of 0.1, 0.3, 0.5, 0.7 are shown in Figure 15.

The optimum objective functions (dynamic compliance) at the aforementioned volume fractions are provided in Table 4. It is interesting to note that relatively slight reduction in optimum dynamic compliance has been achieved (14%) by increasing volume fraction from 0.3 to 0.5 (60%).

**Figure 16.**
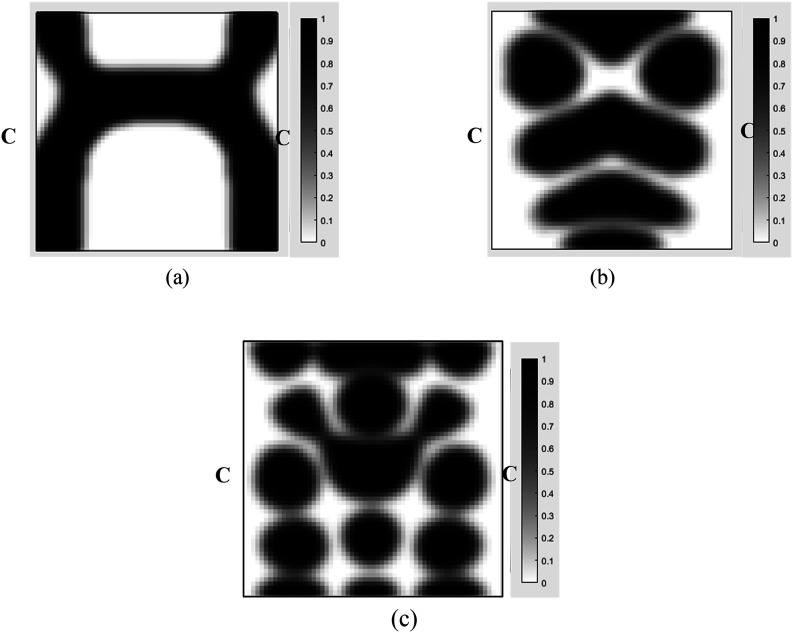
Optimal topology of the MRE layer MRE sandwich plate (CFCF) under different loading frequencies: (a) 45 Hz; (b) 280 Hz; (c) 440 Hz (Case I).

The effect of loading excitation frequency on the optimum topology of the MRE sandwich plate is also investigated and results are shown in Figure 16. It is noted that the magnitude of the applied forces is kept constant at 1000 N and volume fraction is set at 0.5.

**Table 4.** The effect of volume fraction constraints on optimum dynamic compliance for MRE sandwich plate (CFCF) for Case I.

Volume fraction	Optimum dynamic compliance (N.m)
0.1	7.70
0.3	4.95
0.5	4.24
0.7	3.50

**Figure 17.**
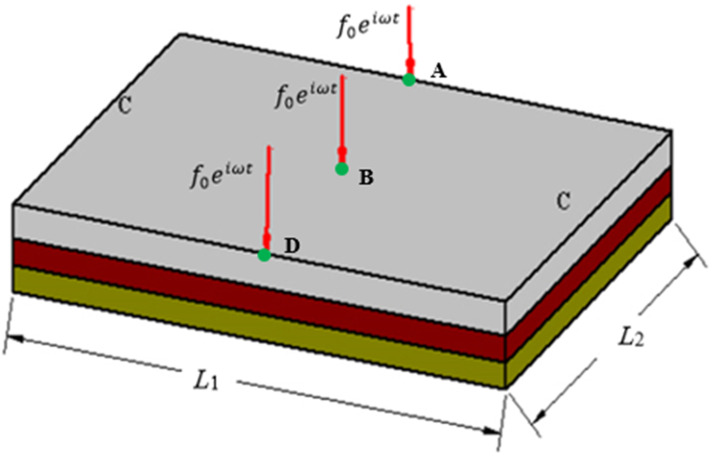
MRE sandwich plate (CFCF) with loading positions at points A, B and D (Case II).

Case II In this case, the MRE sandwich plate (CFCF) is under three concentrated harmonic loadings at points A, B and D as shown in Figure 17 with a magnitude of 1000 N and a frequency of 280 Hz similar to Case I.

**Figure 18.**
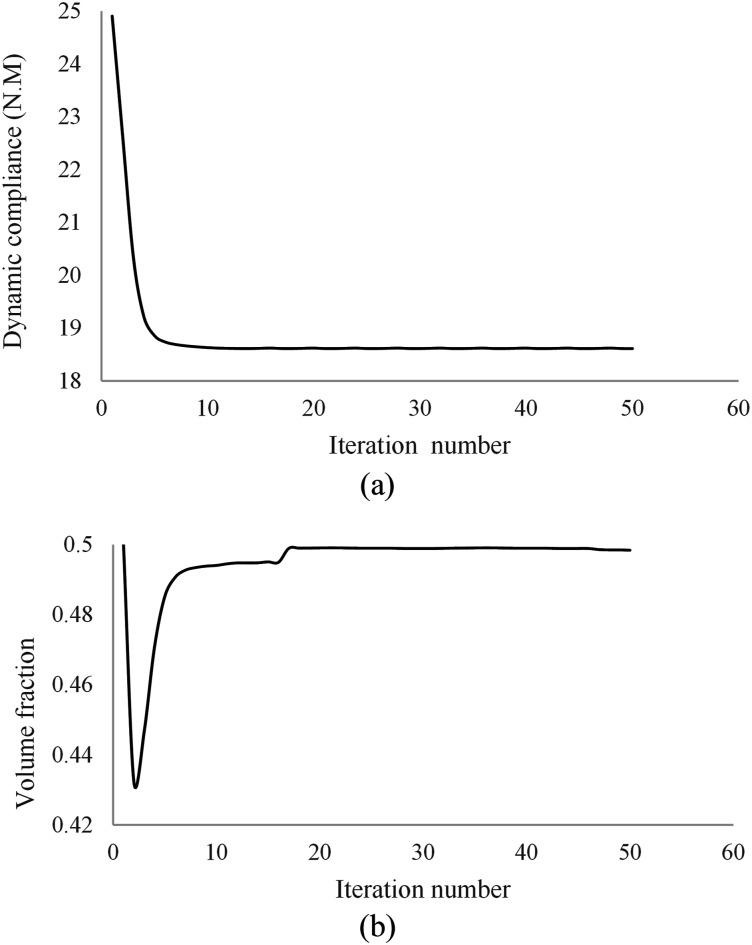
Optimization process history of MRE sandwich plate (CFCF): (a) Convergence history of the dynamic compliance, (b) Convergence history of the volume fraction (Case II).

Similar investigation has been conducted for this case and the developed topology optimization algorithm rapidly converged to the optimum solution as shown in Figure 18. Results show that the dynamic compliance has decreased by 25.2%, from f=24.90 N.m at the initial distribution to f=18.62 N.m at the optimum topology configuration.

**Figure 19.**
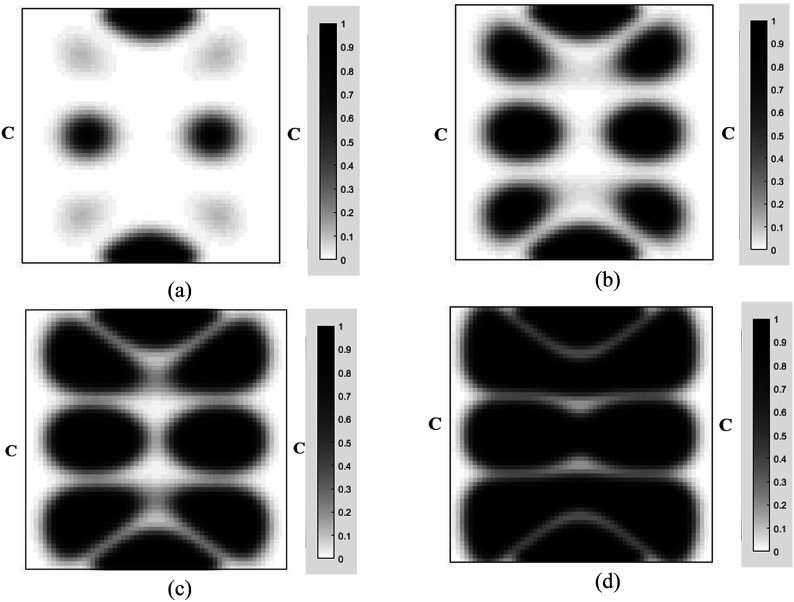
Optimal topology of the MRE layer for MRE sandwich plate (CFCF) under different volume constraints: (a) 0.1; (b) 0.3 (c) 0.5 (d) 0.7 (Case II).

The effect of the volume fraction constraint on the optimal MRE topology has been explored, and the results for volume fractions of 0.1, 0.3, 0.5, and 0.7 are presented in Figure 19.

The optimum objective functions for the aforementioned particular volume fractions are also outlined in Table 5. Results show that despite a substantial increase in the volume fraction from 0.1 to 0.7 (86%), there is only a 34.4% decrease in optimal dynamic compliance.

**Figure 20.**
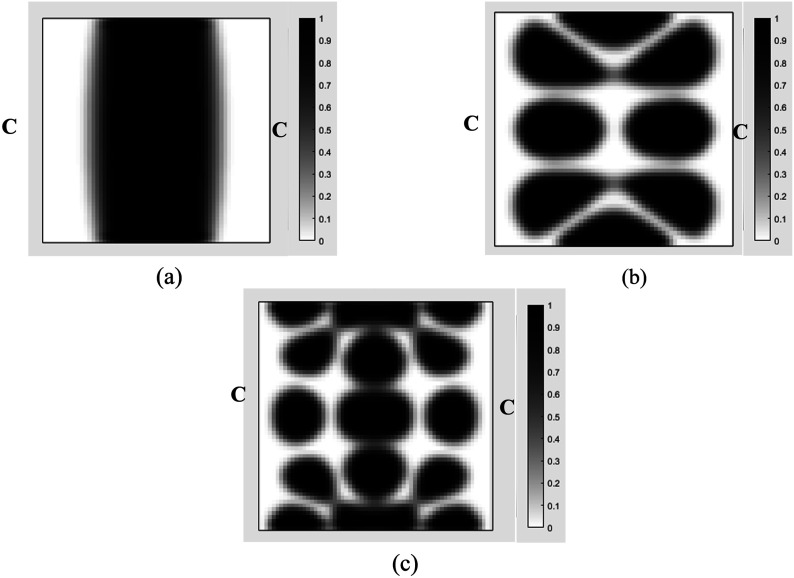
Optimal topology of the MRE layer MRE sandwich plate (CFCF) under different loading frequency: (a) 45 Hz; (b) 280 Hz; (c) 440 Hz (Case II).

Results for the optimum topology of the MRE layer for Case II under different loading frequencies while the amplitude is kept constant at 1000 N are also shown in Figure 20. It is noted that volume fraction is set at 0.5.

Optimum topology results in Figures 16 and 20 show the significant variations in optimal layouts of the MRE layer. These changes occur because different orders of eigenmodes are excited at different frequencies, resulting in different patterns for reducing the compliance. Furthermore, examination of results in Figures 15 and 19 shows that the optimum topology evolves, and some isolated regions become connected as the volume fraction constraint on MRE increases.

**Table 5.** The effect of volume fraction constraints on optimum dynamic compliance for MRE sandwich plate (CFCF) for Case II.

Volume fraction	Optimum dynamic compliance (N.m)
0.1	27.31
0.3	21.16
0.5	18.78
0.7	17.91

## Conclusions and discussions

In this study, a density-based design optimization methodology has been developed to identify the optimum topology of MRE layer in the core of a sandwich plate to maximize its vibration suppression capability. A finite element method has been formulated to evaluate the dynamic response of the sandwich plate and its dynamic compliance. The density of the MRE core layer in each element are considered as the design variables. The SIMP approach is expanded to include material properties interpolation in the MRE layer for the sandwich plate. The method of moving symptoms (MMA) is effectively utilized to identify the optimum layout of the MRE layer. The design optimization methodology is first validated and then effectively used to investigate the optimum topology of optimal topologies of the MRE core layer in MRE-based sandwich plates under various boundary and loading conditions as well as different values of volume fraction of the MRE layer.

## Appendix A

The elemental stiffness and mass matrices are defined as:

(A.1)
$$K^{e}=\overset{3}{\underset{i=1}{\sum }}\left(K_{Ti}+K_{bi}+K_{sM}\right)M^{e}=\overset{3}{\underset{i=1}{\sum }}\left(m_{Ti}+m_{bi}\right)$$

where, $K_{Ti}$ and $K_{bi}$ represent the extensional and bending stiffness matrices associated with the *i*th layer, defined respectively as:

(A.2)
$$K_{Ti}=h_{i}\int_{A}{B_{Ti}}^{T}D_{Ti}B_{Ti}dAK_{bi}=h_{i}\int_{A}{B_{bi}}^{T}D_{bi}B_{bi}dA$$

where,

(A.3)
$$B_{Tc}={\left[\begin{array}{lll} \frac{\partial N_{1}}{\partial x} & \frac{\partial N_{2}}{\partial y} & \frac{\partial N_{1}}{\partial y}+\frac{\partial N_{2}}{\partial x} \end{array}\right]}^{T}B_{Tm}={\left[\begin{array}{lll} \frac{\partial N_{8}}{\partial x} & \frac{\partial N_{9}}{\partial y} & \frac{\partial N_{8}}{\partial y}+\frac{\partial N_{9}}{\partial x} \end{array}\right]}^{T}B_{Tb}={\left[\begin{array}{lll} \frac{\partial N_{3}}{\partial x} & \frac{\partial N_{4}}{\partial y} & \frac{\partial N_{3}}{\partial y}+\frac{\partial N_{4}}{\partial x} \end{array}\right]}^{T}$$

It is noted that the indexes *c*, *m* and *b* are used to represent the constraining, MRE and the basic layers, respectively. And for the bending term the associated matrix is yield as:

(A.4)
$$B_{bi}={\left[\begin{array}{lll} \frac{\partial^{2}N_{5}}{\partial x^{2}} & \frac{\partial^{2}N_{5}}{\partial y^{2}} & 2\frac{\partial^{2}N_{5}}{\partial xy} \end{array}\right]}^{T}$$

Also, $K_{sM}$ signifying the shear stiffness matrix of the MRE layer, defined as:

(A.5)
$$K_{sM}=h_{m}\int_{A}{B_{sm}}^{T}GB_{sm}dAB_{sm}=\left[\begin{array}{l} N_{10}\\ N_{11} \end{array}\right]$$

Similarly, the extension and bending terms for the elemental mass matrix, denoted as $m_{Ti}$ and $m_{bi}$ respectively, are obtained as:

(A.6)
$$m_{Tc}=\rho_{c}h_{c}\int_{A}\left({N_{1}}^{T}N_{1}+{N_{2}}^{T}N_{2}\right)dAm_{Tb}=\rho_{b}h_{c}\int_{A}\left({N_{3}}^{T}N_{3}+{N_{4}}^{T}N_{4}\right)dAm_{Tm}=\rho_{m}h_{c}\int_{A}\left({N_{8}}^{T}N_{8}+{N_{9}}^{T}N_{9}\right)dAm_{bi}=\rho_{i}h_{i}\int_{A}{N_{5}}^{T}N_{5}dA$$
